# Beninese Plant Extracts with Antiplasmodial Activity Select New Allele Variants *Msp*1 and *Msp2* in *Plasmodium falciparum*

**DOI:** 10.1155/2024/9980715

**Published:** 2024-03-20

**Authors:** Hamirath O. Lagnika, Adandé A. Medjigbodo, Oswald Y. Djihinto, Helga M. Saïzonou, Wassiyath A. Mousse, Romaric Akoton, Laurette Djossou, Doris N. Vodounkpe, Latifou Lagnika, Luc S. Djogbénou

**Affiliations:** ^1^Tropical Infectious Diseases Research Centre (TIDRC), University of Abomey-Calavi, 01BP 526 Cotonou, Benin; ^2^Regional Institute of Public Health/University of Abomey-Calavi, BP 384 Ouidah, Benin; ^3^Laboratory of Biochemistry and Bioactive Natural Substances, Unit of Biochemistry and Molecular Biology, University of Abomey-Calavi, 04 BP, 0320 Cotonou, Benin; ^4^Department of Vector Biology, Liverpool School of Tropical Medicine, Pembroke Place, Liverpool L3 5QA, UK

## Abstract

**Background:**

Natural medicinal products are commonly used as a remedy against malaria infections in African populations and have become a major source of information for the screening of new and more effective antiplasmodial molecules. Therefore, *in vitro* studies are needed to validate the efficacy of these medicinal products and to explore the potential effects of such drugs on the genetic diversity of *Plasmodium falciparum*. The current study has investigated the impact of some Beninese plant extracts with antiplasmodial activity on the genetic diversity of *P. falciparum*.

**Method:**

Five (5) ethanolic plant extracts (*Dissotis rotundifolia*, *Ehretia cymosa* Thonn, *Hibiscus surattensis* L., *Cola millenii* K. Shum, and *Costus afer* Ker Gawl) and a compound extracted from *Ehretia cymosa* Thonn (encoded CpE2) were tested against asexual stage parasites of a culture-adapted strain of *P. falciparum*. Subsequently, the *P. falciparum Msp*1 and *Msp*2 markers were genotyped, and the number of allelic variants and the multiplicity of infection (MOI) were compared between drug-exposed and unexposed parasites.

**Results:**

All plant extracts have shown inhibitory activity against asexual *P. falciparum* and selected new allelic variants of the *Msp*1 and *Msp*2 genes compared to unexposed parasites. The newly selected allelic variants were K1_100bp and RO33_300bp of the *Msp*1 gene and FC27_150bp, FC27_300bp, FC27_400bp, and FC27_600bp of the *Msp*2 gene. However, there was no significant difference in MOI between drug-exposed and unexposed parasites.

**Conclusion:**

Our study highlights a source for the selection of new *Msp*1 and *Msp*2 alleles after exposure to antimalarial drugs. These findings pave the way for further studies investigating the true roles of these newly selected alleles in *P. falciparum*.

## 1. Introduction

Malaria is estimated to be directly responsible for 249 million cases with 608000 deaths worldwide in 2022 [1]. African regions are still recording a high burden of this devastating disease with 233 million cases and 580000 deaths, mostly among pregnant women and children under five years of age. Benin is a country belonging to the African region of the World Health Organization (WHO), where malaria has remained a serious threat to the well-being of people. In 2022, 5120164 cases and 2955 deaths due to malaria have been recorded in this country, with 13352864 individuals at risk of malaria infection (38.34% malaria prevalence) [1].

The most prevalent and pathogenic causal agent of malaria infection in African countries is *Plasmodium falciparum* [1, 2]. This malaria parasite is consistently transmitted and maintained in human populations through their definitive host, the female *Anopheles* mosquito [3]. For malaria control strategies targeting *Plasmodium* parasites, the WHO has recommended the use of pyrimethamine-sulfadoxine in pregnant women, seasonal chemoprevention in children under 5 years of age, artemisinin-based combination therapy (ACT) in uncomplicated malaria [4], and, recently, vaccines (RTS, S/AS_01_; R21/Matrix-M) administration for malaria prevention in children [5, 6]. On the other hand, several medicinal plants are commonly used to treat malaria infection within African human populations [7–9]. For example, a study conducted in southern Benin has reported that medicinal plant species such as *Dissotis rotundifolia*, *Ehretia cymosa* Thonn, *Hibiscus surattensis* L., *Cola millenii* K. Shum, and *Costus afer* Ker Gawl are commonly used by populations for malaria treatment [10]. Furthermore, the use of these medicinal plants as malaria remedies has been validated by *in vitro* evaluation of their antiplasmodial activity [11–14].

However, malaria remains a crucial public health concern in endemic countries due in part to the occurrence, emergence, and spread of *Plasmodium* isolates that develop resistance to synthetic antimalarial drugs [15]. Furthermore, *P. falciparum* has been shown to attempt to escape antimalarial drug pressure by diversifying their genome and switching off expression of drug targets [16]. More recently, Greyling et al. reported a slightly positive association between the loss of efficacy (increased IC_50_ values) of some lead antimalarial candidates and the genetic complexity of clinical *P. falciparum* isolates [17].

Today, all malaria researchers are joining their efforts to discover new, more effective, safe, and inexpensive antimalarial drugs to combat drug resistance in *Plasmodium* parasites. For this purpose, medicinal plants with antiplasmodial properties have become valuable sources to screen for new potential antiplasmodial compounds against resistant *P. falciparum* [18–20]. Therefore, it is crucial to evaluate the antiplasmodial efficacy of these traditional medicines and assess their likely effects on the genetic diversity of *P. falciparum*.

Although natural products have been used intensively in African countries as antiplasmodial medicines, so far, no studies have investigated the impact of these plant products on the genetic diversity of *P. falciparum*. In this paper, we exposed *P. falciparum* parasites to different concentrations of some Beninese plant extracts with proven antiplasmodial activity. Subsequently, the drug-exposed and unexposed parasites were genotyped using *Msp*1 and *Msp*2 markers.

## 2. Materials and Methods

### 2.1. Asexual *P. falciparum* Drug Sensitivity Assays (DSAs)

#### 2.1.1. Description of Screened Plant Extracts

The crude ethanolic extracts of four (4) Beninese medicinal plants including *Dissotis rotundifolia*, *Hibiscus surattensis* L., *Cola millenii* K. Shum, and *Costus afer* Ker Gawl, which have been previously found to be active against asexual *P. falciparum* [11–14], were used. Furthermore, the crude extract of the antimalarial plant species *Ehretia cymosa* Thonn and a compound encoded CpE2, previously extracted from this plant (unpublished data), was screened. Collection and identification of plant materials, as well as preparation of crude extracts, have been described in our previous studies [11–14]. In the current study, the crude extracts were dissolved in 1% dimethyl sulfoxide (DMSO) to prepare the tested concentrations.

#### 2.1.2. Culture of P. falciparum

A strain of *P. falciparum* called Ben229 already adapted to routine *in vitro* culture at the Tropical Infectious Diseases Research Centre (TIDRC) of the University of Abomey-Calavi, Benin, was thawed and established in *in vitro* culture following standard protocols [21, 22] with minor modifications. Details on how Ben229 has been adapted to routine *in vitro* culturing are available on the Patentscope website of the World Intellectual Property Organization (WIPO) under reference no. WO2023/180790 A1 [23].

Briefly, parasites were cultured at 4% hematocrit (O + red blood cells (RBCs)) in complete medium (RPMI-1640 containing 2 mM L-glutamine, 25 mM HEPES, 0.85 g/L sodium bicarbonate supplemented with 50 mg/L hypoxanthine (Gibco), and 0.25% (*w*/*v*) Albumax II (Gibco) as human AB^+^ serum substitute [24] without gentamycin). Cell culture flasks (T-75 cm^2^ Nunclon™, Denmark) were provided with 3% O_2_, 5% CO_2_, and 92% N_2_ (AIR LIQUIDE France Industrie) and incubated in a CO_2_ incubator settled at 37°C.

#### 2.1.3. Synchronization of P. falciparum Ring Stage

Double synchronized ring stage parasites were obtained by treating a culture containing approximately 8% parasitemia with a solution of 5% D-sorbitol (Sigma). Two days after synchronization, the ring stage parasites were seeded at 1% parasitemia for the drug sensitivity assays.

#### 2.1.4. In Vitro Drug Sensitivity Assay Procedures

A protocol similar to that described in [25] with some modifications was used to determine the inhibitory effects of plant extracts on asexual parasites of *P. falciparum*. Briefly, a 96-well tissue culture plate was filled with three (3) replicates of 100 *μ*L of each plant extract at 0.39, 1.56, 6.25, 25, and 100 *μ*g/mL. Complete untreated parasite medium (CPM) was used as a negative control, and 100 *μ*g/mL artesunate (Artesun® injectable, Fosun Pharma) was used as a positive control. Furthermore, 100 microliters (100 *μ*L) of double synchronized parasite culture was added at 1% parasitemia and 4% hematocrit in each well, and the plate was placed in an incubator. The chamber was gassed for 6 minutes with mixed gas (3% O_2_, 5% CO_2_, and 92% N_2_ (AIR LIQUIDE France Industrie)) and incubated at 37°C for 72 h. After the incubation period, thin film smears were prepared from each well. The smears were fixed with absolute methanol and stained with 10% Giemsa for 20 min. The smears were observed under a light microscope using a 100x oil immersion objective lens, and antiplasmodial activity of each plant extract has been assessed by determining parasite density. The DSAs were repeated three times.

Plant extract activity was classified according to the IC_50_ values as follows: “good” (IC_50_ < 10 *μ*g/mL), “moderate” (IC_50_: 10 *μ*g/mL to 50 *μ*g/mL), “low” (IC_50_: 50 *μ*g/mL to 100 *μ*g/mL), and “inactive” (IC_50_ > 100 *μ*g/mL) [26]. The percentage (%) of inhibition of asexual parasite growth was calculated for each concentration of plant extract concentration as described in [27] following the formula (1–(mean parasitaemia from the triplicate of each concentration/mean parasitaemia from the triplicate of negative control)) × 100.

### 2.2. Evaluation of the Genetic Diversity of *P. falciparum Msp*1 and *Msp2*

#### 2.2.1. DNA Extraction

Subsequently, DNA from the remaining parasite cultures in each plate well was extracted using Chelex 100® (Bio-Rad Laboratories, CA, USA) as previously described [28]. DNA extracts were stored at -20°C until amplification reaction.

#### 2.2.2. Genotyping of the *P. falciparum Msp*1 and *Msp*2 Genes

A protocol previously described [29, 30] was used to amplify the *Msp*1 and *Msp*2 genes of *P. falciparum*. Polymorphic allelic families of the *Msp*1 gene (K1, Mad20, and RO33) and *Msp*2 gene (FC27 and 3D7) were amplified by nested PCR amplification. Briefly, primary amplification used 0.25 *μ*L of each of the primers corresponding to the conserved regions of block 2 *PfMsp*1 such as M1-OF/M1-OR and block 3 *PfMsp*2 such as M2-OF/M2-OR in the presence of 3.55 *μ*L of water, 5 *μ*L of buffer 5x, 4 *μ*L of 25 mM MgCl_2_, 1 *μ*L of 5 mM dNTP, 0.25 *μ*L of Taq DNA Polymerase (BioLabs® Inc.), and 5 *μ*L of DNA extract. For the secondary PCR reaction, primer pairs specific to each allelic family of *PfMsp*1 and *PfMsp*2 were used under the same conditions as for the first PCR round, but with different reaction volumes. The primer pairs used were K1/K2 for the K1 family, Mad20-1/Mad20-2 for the Mad20 family, and RO33-1/RO33-2 for the RO33 family of *PfMsp*1. For the *PfMsp*2 allelic families, the primer pairs FC27-1/FC27-2 and 3D7-1/3D7-2 were used for the FC27 and 3D7 families, respectively. Cycle conditions for both rounds of PCR reactions were an initial denaturation at 94°C for 5 min followed by 35 cycles of 94°C for 30 s, annealing at 55°C for 1 min, and extension at 72°C for 1 min, with a final extension at 72°C for 5 min. The size of the amplification products was assessed by 2% agarose gel electrophoresis and UV visualization after ethidium bromide staining.

The multiplicity of infection (MOI) and the mean number of allelic families of the *Msp*1 (K1, Mad20, and RO33) and *Msp*2 (3D7 and FC27) genes were determined in exposed and unexposed parasites. The following formula was used to calculate the

(1) $$\begin{array}{c} \text{MOI}:\frac{\text{Total number of alleles detected for }Msp1\text{ and }Msp2\text{ genes}}{\text{Total number of positive samples}.}. \end{array}$$

### 2.3. Statistical Analysis

For microscopic assays, parasite density was determined for each thin smear by counting all stages of asexual parasites against 5000 RBCs. Data were transformed, normalized, and subjected to a nonfit linear regression test with variable slope (log (inhibitor) vs. normalized response test) in GraphPad Prism version 8.02 (San Diego, California, USA), to allow estimation of 50% inhibitory concentrations (IC_50_) for each plant extract. The significance threshold was established at *p* < 0.05.

## 3. Results

### 3.1. Inhibition of the Growth of Sexual Parasites

**Figure 1:**
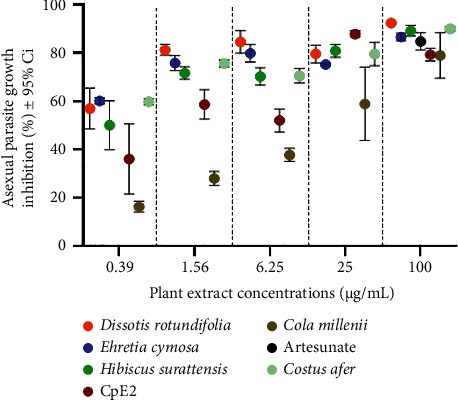
Asexual *P. falciparum* growth inhibition by the plant extracts.

Fifty (50) % inhibitory concentrations (IC_50_) estimated for *Dissotis rotundifolia*, *Ehretia cymosa* Thonn, *Hibiscus surattensis* L., *Cola millenii* K. Shum, *Costus afer* Ker Gawl, and CpE2 were 0.09 ± 0.35 *μ*g/mL, 0.03 ± 0.22 *μ*g/mL, 0.27 ± 0.34 *μ*g/mL, 11.63 ± 0.53 *μ*g/mL, 0.06 ± 0.24 *μ*g/mL, and 1.36 ± 0.53 *μ*g/mL, respectively (Figure 1). As expected, all plant extracts exhibited good antiplasmodial activity, except *Cola millenii* K. Shum, which showed moderate activity against asexual *P. falciparum*. However, all these plant extracts have exhibited more than 75% inhibition of parasite growth at 100 *μ*g/mL similarly to the positive control artesunate (Figure 1). *Dissotis rotundifolia*, *Ehretia cymosa* Thonn, *Hibiscus surattensis* L., *Cola millenii* K. Shum, *Costus afer* Ker Gawl, CpE2, and artesunate showed a growth inhibition of *P. falciparum* of 92.39%, 86.66%, 89.22%, 79%, 90%, 79.33, and 84.83%, respectively (Figure 1). The crude plant extracts *Dissotis rotundifolia*, *Ehretia cymosa* Thonn, *Hibiscus surattensis* L., and *Costus afer* Ker Gawl showed more effective antiplasmodial activity than the reference antimalarial drug artesunate. Subsequently, the exposed and unexposed parasites were genotyped to determine the genetic diversity of the *Msp*1 and *Msp*2 genes.

### 3.2. Effects of Plant Extract Exposures on Genetic Diversity at *Msp*1 and *Msp*2 Loci in Asexual *P. falciparum*

**Figure 2:**
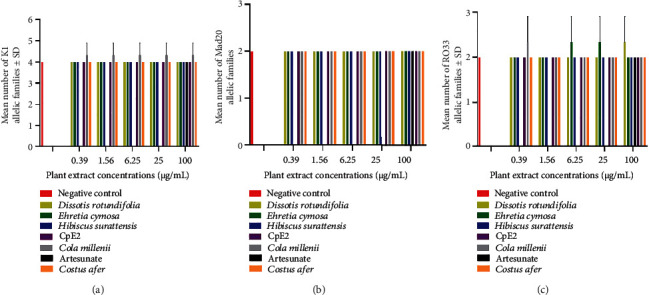
*Cola millenii* selected new alleles of both *Msp*1_K1 and *Msp*1_RO33 allelic families while *Ehretia cymosa*, *Dissotis rotundifolia*, and *Cola millenii* induced the apparition of new alleles of *Msp*1_RO33, in asexual *P. falciparum*. (a), (b), and (c) show the mean numbers of K1, Mad20, and RO33 allelic families of *Msp*1 gene, respectively.

**Figure 3:**
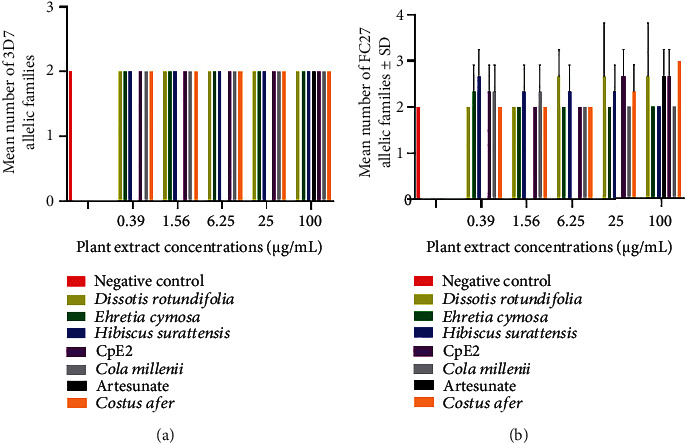
All plant extracts selected new alleles of *Msp*2-FC27 allelic family in asexual *P. falciparum*. (a) and (b) show the mean numbers of 3D7 and FC27 allelic families of *Msp*2 gene, respectively.

Plant extracts have inhibited the growth of parasites but have selected new alleles of the *Msp*1 and *Msp*2 genes. In unexposed parasites, the number of distinct alleles identified in the *Msp*1 gene was 4 for K1 (150, 200, 250, and 700 bp), 2 for Mad20 (150 and 250 bp), and 2 for RO33 (200 and 700 bp) (Table 1). For the *Msp*2 gene, 2 different alleles were identified for 3D7 (250 and 350 bp) as well as for FC27 (180 and 900 bp) (Table 1), while new alleles K1_100bp and RO33_300bp of the *Msp*1 gene (Table 1 and Figure 2) and new alleles FC27_150bp, FC27_300bp, FC27_400bp, and FC27_600bp of the *Msp*2 gene (Table 1 and Figure 3) were observed in parasites exposed to natural antimalarial products. The new *Msp*1 allelic variants have been selected in parasites exposed to *Cola millenii* K. Shum (K1_100bp and RO33_300bp), *Ehretia cymosa* Thonn (RO33_300bp), and *Dissotis rotundifolia* (RO33_300bp), while all plant extracts, as well as artesunate, have selected new alleles of the *Msp*2-FC27 allelic family (Figure 3 and Table 1). However, no significant differences (Mann–Whitney test, *p* > 0.05) were observed between the mean numbers of alleles recorded in parasites exposed to antimalarial drugs and unexposed ones. Furthermore, the allelic families *Msp*1_Mad20 and *Msp*2_3D7 have not shown a new variant even exposed to antimalarial drugs. Although new *Msp*1 and *Msp*2 alleles were recorded in drug-exposed parasites, no significant impact was observed on the MOI of the *P. falciparum Msp*1 and *Msp*2 genes (Mann–Whitney test, *p* > 0.05) (Table 2).

## 4. Discussion

The complexity of infection in malaria-endemic areas is exacerbated by the presence of genetically diverse *Plasmodium falciparum* strains that represent a risk of spreading more virulent or drug-resistant malaria parasites. Genetic diversity in malaria parasites could be driven by pressures from antimalarial drugs as they function by targeting specific parasite loci. The main aim of the current work was to investigate the effect of Beninese plant extracts with antiplasmodial activity on *P. falciparum* genetic diversity using length polymorphic markers such as merozoite surface proteins coding genes *Msp*1 and *Msp*2.

As for artesunate, all tested plant extracts have shown inhibitory activity in *P. falciparum*, confirming the use of these medicinal plants as antimalarial drugs in Benin. In fact, *Dissotis rotundifolia*, *Ehretia cymosa* Thonn, *Hibiscus surattensis* L., and *Costus afer* Ker Gawl showed more effective antiplasmodial activity than the reference antimalarial drug artesunate. This could be explained by the fact that crude extracts have multiple metabolites or constituents that could have antiplasmodial activity. Therefore, the crude extract with several antiplasmodial compounds results in a highly effective product compared to artesunate, which is a single active molecule.

Since antimalarial drugs are generally isolated from natural plant extracts [20], the plant extracts of this study could serve as the basis for the development of new more effective drugs against *P. falciparum*. It is therefore crucial to monitor potential factors that could affect important phenotypes, including drug resistance, virulence, growth rate, and transmissibility in antimalarial drug-exposed parasites. In the current work, we observed the occurrence of new allelic variants of K1, RO33, and FC27, respectively, in the *Msp*1 and *Msp*2 genes in *P. falciparum* malaria parasites exposed to antimalarial plant extracts. This selection of new alleles in exposed parasites could be related to the pharmacokinetics of plant extracts. Antimalarial drugs are known to use metabolic and elimination pathways that are prone to significant genetic variations in targeted parasites [17, 31]. Such genetic variations could lead to the selection of new alleles in drug-exposed *P. falciparum* parasites. As a result, these newly selected alleles could imply new phenotypes in these malaria parasites. Using a high-throughput single nucleotide polymorphism (SNP) genotyping, researchers have highlighted the genetic diversity underlying some traits of interest, such as resistance against antimalarial compounds in *P. falciparum* [16]. Indeed, following chloroquine diphosphate exposure, high genetic diversity near 460 kb was reported at the chloroquine resistance transporter (*Pf*crt) locus of *P. falciparum* in chloroquine sensitive isolates of *P. falciparum*. This positive selection points out a likely linkage disequilibrium (LD) between genes known to be subjected to strong diversity in *P. falciparum* and antimalarial resistance genes. Alternatively, the new *Msp*1 and *Msp*2 alleles found in *P. falciparum* exposed to plant extracts could have some impacts on the parasites' life history traits. Therefore, fundamental studies are required to investigate the relationship between the selected *Msp*1 and *Msp*2 alleles and the key phenotypic characteristics in *P. falciparum*, including the cell development of asexual blood stage parasites, virulence, the ability to survive drug exposure, and their infectivity to the main malaria vector *Anopheles gambiae*.

Although new alleles were observed in exposed *P. falciparum* parasites, plant extracts have not induced a significant impact on the MOI of the *PfMsp*1 and *PfMsp*2 genes. A similar level of MOI found in nonexposed and exposed parasites indicates that newly selected alleles did not always result from a recombination between genetically different parasite lines during the parasite sporogonic life cycle, contrasting the assumption that new *P*. *falciparum* clones selected following drug treatments are inoculated in humans by infected *Anopheles* mosquitoes [32, 33]. Further research is needed to ascertain the origin of new alleles detected in *P. falciparum* parasites after exposure to antimalarial drugs.

Furthermore, our findings indicated that, in *P. falciparum* parasites exposed to plant extracts, only the allelic families *Msp*1_K1/RO33 and *Msp*2_FC27 showed new alleles (K1_100bp and RO33_300bp for *Msp*1 and FC27_150bp, FC27_300bp, FC27_400bp, and FC27_600bp for *Msp*2). The response of *P. falciparum* parasites when exposed to antimalarial plant extracts could be related to the specific polymorphic region targeted by specific extract constituents or/and the degree of polymorphism of the targeted gene. Antiplasmodial drug exposures would select several allelic variants in a highly polymorphic (block 3) region of the *Msp*2 gene than the less polymorphic (*Msp*1 gene) region, as the *Msp*2 gene is commonly exploited as vaccine target [34] and known to be responsible for *P. falciparum* drug resistance strategies [35]. Indeed, Engelbrecht et al. [36] have reported that parasites carrying FC27-like genotypes of the *Msp*2 gene were twice as likely to be found in cases of symptomatic malaria treated with antimalarial drugs than in asymptomatic controls without treatment. In addition, FC27 alleles of the *Msp*2 gene were shown to become more prevalent than 3D7 during and after a peak incidence of malaria [37]. Collectively, the FC27 alleles of the *Msp*2 gene would probably play a critical role in *P. falciparum* parasites exposed to stressors such as antiplasmodial drugs and the host's immune response. A better understanding of the role played by these genetic markers in *P. falciparum* could provide new insights to advance the development of most efficient plant-derived antimalarial compounds.

## 5. Conclusions

The current work reports the selection of new *Msp*1 and *Msp*2 alleles in *P. falciparum* parasites exposed to natural antiplasmodial products. These findings highlight the necessity to implement periodic molecular monitoring of the potential effects of antimalarial products on the biology of *P. falciparum*.

## Figures and Tables

**Table 1:** *Msp*1 and *Msp*2 alleles of *P. falciparum* detected in exposed and unexposed parasites.

Genes	Allelic families	Fragment sizes (base pairs) per plant extract treatment							
		Negative control	*Dissotis rotundifolia*	*Ehretia cymosa*	*Hibiscus surattensis*	CpE2	*Cola millenii*	Artesunate (positive control)	*Costus afer*
*Msp*1	K1	150	150	150	150	150	100	150	150
		200	200	200	200	200	150	200	200
		250	250	250	250	250	200	250	250
		700	700	700	700	700	250	700	700
							700		
	Mad20	150	150	150	150	150	150	150	150
		250	250	250	250	250	250	250	250
	RO33	200	200	200	200	200	200	200	200
		700	300	300	700	700	300	700	700
			700	700			700		

*Msp*2	3D7	250	250	250	250	250	250	250	250
		350	350	350	350	350	350	350	350
	FC27	180	150	180	180	180	180	180	180
		900	180	300	300	300	300	400	300
			300	900	400	400	900	900	400
			400		900	900			600
			800						900
			900						

Values shown in the table represent the different alleles classified according to the length (in base pairs).

**Table 2:** Multiplicity of infection (MOI) in the allelic families K1, Mad20, RO33, 3D7, and FC27.

Genes	Allelic families	MOI							
		Negative control	Artesunate (positive control)	*Dissotis rotundifolia*	*Ehretia cymosa*	*Hibiscus surattensis*	*Cola millenii*	*Costus afer*	CpE2
*Msp*1	K1	4	4	4	4	4	4.33	4	4
	Mad20	2	2	2	2	2	2	2	2
	RO33	2	2	2.07	2.13	2	2.07	2	2

*Msp*2	3D7	2	2	2	2	2	2	2	2
	FC27	2	2.67	2.4	2.07	2.33	2.13	2.27	2.33

## Data Availability

Data supporting the results of our study will be freely made available to readers on request to corresponding author.
